# Abnormal Whole Blood Thrombi in Humans with Inherited Platelet Receptor Defects

**DOI:** 10.1371/journal.pone.0052878

**Published:** 2012-12-28

**Authors:** Francis J. Castellino, Zhong Liang, Patrick K. Davis, Rashna D. Balsara, Harsha Musunuru, Deborah L. Donahue, Denise L. Smith, Mayra J. Sandoval-Cooper, Victoria A. Ploplis, Mark Walsh

**Affiliations:** 1 W. M. Keck Center for Transgene Research, University of Notre Dame, Notre Dame, Indiana, United States of America; 2 Department of Emergency Medicine, Memorial Hospital of South Bend, South Bend, Indiana, United States of America; University of Leuven, Belgium

## Abstract

To delineate the critical features of platelets required for formation and stability of thrombi, thromboelastography and platelet aggregation measurements were employed on whole blood of normal patients and of those with Bernard-Soulier Syndrome (BSS) and Glanzmann’s Thrombasthenia (GT). We found that separation of platelet activation, as assessed by platelet aggregation, from that needed to form viscoelastic stable whole blood thrombi, occurred. In normal human blood, ristocetin and collagen aggregated platelets, but did not induce strong viscoelastic thrombi. However, ADP, arachidonic acid, thrombin, and protease-activated-receptor-1 and -4 agonists, stimulated both processes. During this study, we identified the genetic basis of a very rare double heterozygous GP1b deficiency in a BSS patient, along with a new homozygous GP1b inactivating mutation in another BSS patient. In BSS whole blood, ADP responsiveness, as measured by thrombus strength, was diminished, while ADP-induced platelet aggregation was normal. Further, the platelets of 3 additional GT patients showed very weak whole blood platelet aggregation toward the above agonists and provided whole blood thrombi of very low viscoelastic strength. These results indicate that measurements of platelet counts and platelet aggregability do not necessarily correlate with generation of stable thrombi, a potentially significant feature in patient clinical outcomes.

## Introduction

An initial step in thrombus formation in the injured vascular endothelium is the adhesion of platelets to exposed subendothelial components, e.g., von Willebrand Factor (vWF), under high rates of shear, via the interaction of the platelet glycoprotein (GP) 1b/V/IX receptor complex with subendothelial vWF [1]. This tethering of platelets then promotes their firmer binding to subendothelial collagen (COL) fibers via platelet receptors, e.g., GPVI [2], [3] and integrin αIIβ1 [4]. During this process, platelets are activated, leading to platelet shape changes, aggregation, release of aggregation agonists, e.g., ADP and Ca^2+^, from dense granules, and release of other biologically active agents, e.g, growth factors, hemostasis agents, and adhesion proteins, from α-granules [5]. The elevation of intracellular Ca^2+^ results in enzymatic liberation of arachidonic acid (AA) from phospholipids, subsequently forming thromboxane A2 (TxA2), which induces platelet activation. Other signaling events occur as a result of agonist-platelet receptor interactions, one example being ADP interactions with its platelet receptor, P2Y_12_ [6], which results in activation of the integrin complex, αIIb/β3, the major fibrinogen receptor on platelets [7]. This step promotes platelet aggregation via fibrinogen bridging and thrombus growth.

Discrete steps of platelet activation have been studied in vitro by exogenous activators, e.g., ADP and AA. Additional studies employing gene-altered mice and patients with specific platelet dysfunctions, e.g., GPIb defects in Bernard Soulier Syndrome (BSS) [8], and αIIb/β3 abnormalities in Glanzmann’s Thrombasthenia (GT), have also been employed to establish mechanisms of platelet function. We propose that additional significant advances can be made by employing the critical end-point of whole blood thrombus-based examination of platelet function, in combination with platelet aggregation studies, which would expand knowledge on relationships between the receptor interactions leading to platelet aggregation and stable thrombus formation. We undertook such a study, with the aid of blood from very rare BSS and GT patients. The results of this investigation are reported herein.

## Materials and Methods

### Blood Collection

This study was designed to be consistent with the US CFR and ICH Guidelines on Good Clinical Practices. Venous human blood was collected by licensed phlebotomists from 10 normal males and females, two female BSS patients (BSS-1 and BSS-2), both heterozygous parents of one of the BSS (BSS-1M and BSS-2F) patients, one male GT (GT-1) patient, 2 female GT patients (GT-2 and GT-3), and the heterozygous mothers of the two female GT patients (GT-2M and GT-3M), none of whom reported interfering medications or recent platelet infusions. Polystyrene vacutainer tubes, containing either 3.2% sodium citrate (9∶1 v:v), 75 U of Na^+^-heparin, or 1.8 mg EDTA/ml, were used. CBCs and metabolic profiles were rapidly obtained on EDTA-treated blood. PT, aPTT, and fibrinogen levels were determined using Diagnostica Stago (Parsnippany, NJ) STArt kits and a STArt-8 hematology analyzer. IRB approval was obtained from Memorial Hospital of South Bend (MHSB) and informed consent forms were signed by all control and subject patients in accord with the Declaration of Helsinki.

### Blood Smears

Blood smears were fixed on slides with methanol, stained with Volu-Sol (Salt Lake City, UT) Dip Stain, and fixed with Volu-Sol Stain Solution. The washed and air-dried slides were coverslipped with Permount mounting medium (Electron Microscopy Sciences, Hatfield, PA) and examined microscopically. Platelet sizes, measured as the diameter at the longest axis, were acquired from the images. Each image contained 4–10 platelets/field, and 3–10 images/blood smear were obtained.

### Sequencing of WBC DNA

Genomic DNA from WBC was used for PCR cloning of the exons. Traditional Sanger sequencing was accomplished using an ABI 3730×l 96-capillary sequencer with a variety of custom designed primers for the complete exon 2 of the GP1b gene (*GPIBA*), which contains the entire GPIb protein open reading frame (ORF), for BSS-1 and BSS-2. Similarly, to identify the genetic abnormalities in GT-1, primers were designed to amplify all 30 exons of the gene (*ITGA2B*) encoding the ORF of integrin αIIb (CD41) and all 15 exons of the gene (*ITGB3*) coding for the ORF of integrin β3 (CD61), along with the intron flanking regions of each exon to insure integrity of splice sites. 4Peaks software (Amsterdam, The Netherlands) was used for viewing and editing sequence trace files with a Mac OSX 10.6.6 system.

### Clot Retraction

Control and subject blood samples were separately drawn into vacutainer serum tubes and allowed to stand vertically undisturbed for 30 min. A Nikon digital SLR camera suspended by a tripod, captured a picture of the specimen along the transverse plane looking vertically down the tube.

### Flow Cytometric Analysis (FCA)

#### Staining of platelets

Staining of platelets from platelet rich plasma (PRP) was performed according to the protocol from BD Biosciences with slight modifications. Freshly drawn blood in heparin was centrifuged at 80×g for 20 min and the top PRP layer was gently removed and fixed with 2% paraformaldehyde at room temperature for 10 min. The platelets were treated 2× with wash solution (1% FBS/1X PBS) with centrifugation at 750×g for 10 min. After the final wash, the platelets were resuspended in 6 ml of this same solution. An aliquot of 100 µl platelets were utilized to label with rabbit-anti-human β3 antibody (1∶10 dilution) (#AP8672b, Abgent, San Diego, CA). This antibody recognizes the peptide region 740–769 at the C-terminal β3 region of GPIIIa. After 30 min at room temperature, the platelets were washed once and then incubated with donkey-anti-rabbit Alexa Fluor488-conjugated 2° antibody (Molecular Probes, Carlsbad, CA) in the dark for 30 min at room temperature.

Aliquots (100 µl) of fixed platelets were also labeled for different experiments with 0.125 µg each of FITC mouse anti-human αIIb antibody (#555466, BD Pharmingen, San Jose, CA), FITC mouse anti-human β3 antibody (#555753 BD Pharmingen), PE mouse anti-human GPIb antibody (#555473 BD Pharmingen), PE mouse anti-human α2b antibody (integrin β1) (#556049 BD Pharmingen), FITC mouse anti-human GPIa (integrin α2) (BD Pharmingen #555498), PE mouse IgG1 isotype control (#555749 BD Pharmingen), and FITC mouse IgG1 isotype control (#555748, BD Pharmingen). All labeled platelet suspensions platelets were finally washed once, resuspended in 1% paraformaldehyde, and analyzed by FCA.

#### FCA of labeled platelets

A 3 laser 9 color FACSAria III (BD Biosciences, San Jose, CA) was utilized for FCA of the labeled platelets using the FITC and PE channels. Acquisition and analysis were performed by gating on side scatter (SSC-H) and fluorescence (FITC-β3), setting the scales to logarithmic amplification. Cells in suspension were analyzed at a flow rate of 1 ml/sec and 10,000 events were recorded for analysis.

### Thromboelastography (TEG)

For TEG analyses of recalcified whole blood, 20 µl of 0.2 M CaCl_2_ was added to 340 µl of citrated blood. Data were collected for 1.5–2.0 hr. Rapid thromboelastograms were obtained after addition of 20 µl of 0.2 M CaCl_2_ and 10 µl of the Rapid TEG kit mixture (Haemoscope, Niles, IL), containing reconstituted soluble human Tissue Factor (sTF)/kaolin/phospholipid, to 340 µl of citrated blood. Thrombin-enhanced thromboelastograms contained 1 unit thrombin (ERL, South Bend, IN)/20 µl 0.2 M CaCl_2_ and 340 µl of citrated blood. Kaolin-stimulated thromboelastograms were obtained using 1 ml of citrated whole blood and kaolin solution (Haemoscope). Then, 360 µl of blood was placed in the cup with 20 µl of 0.2 M CaCl_2_. To assess the competency of the human fibrinolytic system, 200 ng of human tissue-type plasminogen activator (htPA) ±5 mM ε-aminocaproic acid (EACA), was added to the reaction vessels along with the Ca^2+^/sTF/kaolin sample.

TEG data were collected using an automated Thromboelastograph 5000 (Haemoscope) and analyzed using Haemoscope TAS Version 4.3 Software. The hemostasis parameters obtained were: R, the time from the start of the reaction until the onset of thrombus formation; K_20_, the time from R to a 20-mm clot strength signal; A, the angle in degrees, which reflects the rate of clot formation; MA_max_, the maximum amplitude (mm) attained, which is a reflection of the contributions of crosslinked fibrin and platelets to thrombus strength; and the fibrinolysis parameters, LY_30_ and LY_60_, which are the percent reductions of the area under the thromboelastograms from the time that MA_max_ is attained until 30 and 60 min, respectively, after MA_max_.

### Platelet Functionality in Thrombus Formation Measured by TEG

For TEG-based platelet analyses, 360 µl of the kaolin/blood sample was added to 20 µl of saline/20 µl of 0.2 M CaCl_2_. In a separate reaction cup, 10 µl of the Activator F kit (reptilase+human (h)FXIIIa) was mixed with 360 µl of heparinized blood to obtain the contribution to the MA_max_ of crosslinked fibrin alone (MA_F_). In other assays, 10 µl of Activator F and 360 µl of heparinized blood were employed, and either the recommended 10 µl of the ADP kit solution, 10 µl of the AA kit solution, 12 µl of 10 mg/ml ristocetin (RIST), 10 µl of 100 µg/ml of COL; 12 µl of 1 mM of the protease activated receptor (PAR)-1 agonist, PA-1 (H-SFLLRN-OH; Anaspec, Fremont, CA), or 12 µl of 20 mM PAR-4 agonist, PA-4 (H-GYPGKF-NH_2_; Anaspec), to activate platelets. The MA_max_ for the reaction obtained with each platelet activator - MA_F_ provides MA_P_. The MA_max_ with Ca^2+^/kaolin - MA_F_, yields MA_K_. The % stimulations were calculated as: MA_P_/MA_K_×100.

### Platelet Aggregometry

For human blood, 300 µl saline and 300 µl heparinized blood were mixed in the reaction cup of a Multiplate impedance aggregometer (Verum Diagnostica, Munich, Germany), followed by a 3 min incubation. Then, 20 µl of 0.2 mM ADP, 20 µl of 15 mM AA, 20 µl of 100 µg/ml COL, 50 µl of 10 mg/ml RIST, 20 µl of 1 mM PA-1, or 12 µl of 20 mM PA-4 were added and recordings initiated.

In all cases, measurements were recorded for 6 min and plotted by the software as arbitrary aggregation units (AU) versus time. The areas under each curve, from 0–6 min, were taken as the platelet aggregation response.

## Results

### BSS Patients

Two of these extremely rare patients were identified for study and were diagnosed in other facilities earlier in life.

#### Patient BSS-1

**Figure 1:**
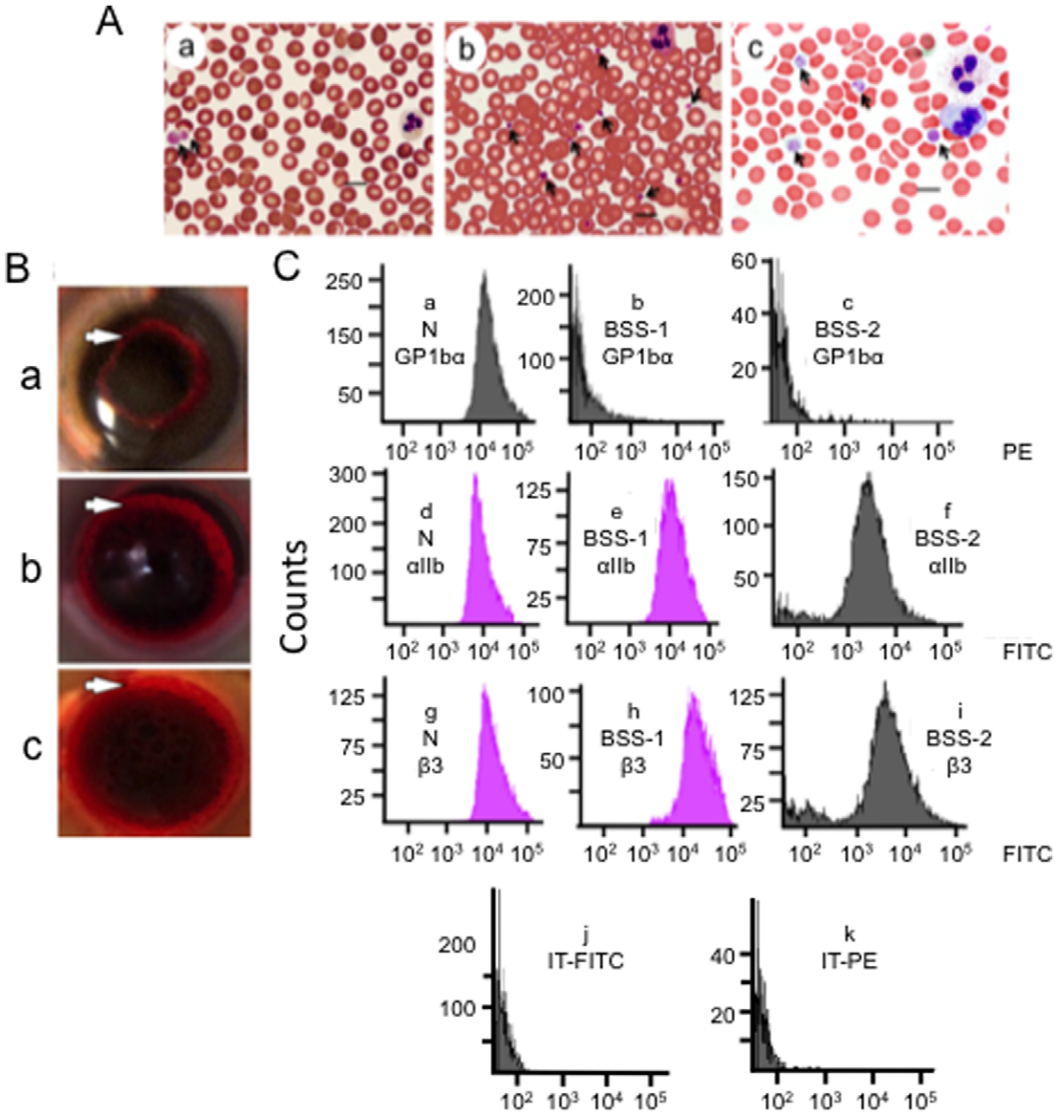
Platelets of BSS patients. (A) Blood smears were stained with Volu-Sol Dip Stain. (a) BSS-1. The platelets (arrows) vary in size and exhibit a granular composition with a fuzzy coat. Giant platelets (>5 µm) are indicated by arrows. (b). Normal blood showing platelets (arrows) at 1–3 µm diameter. (c) BSS-2. Giant platelets (>5 µm) are indicated by arrows. The blood smears were viewed with a Nikon E600 Eclipse microscope containing a Plan Apo VC 100×/1.40 N.A. Oil lens, acquired with a Nikon DS Ri1 color digital camera via Nikon Elements AR 3.2 imaging software, and saved as TIFF files. Measurements of cells were obtained with the measurement tool of the Nikon Elements-AR 3.2 imaging software. The bars on all images represent 10 µm. (B) Whole blood clot retraction of (a) BSS-1, (b) normal human, and (c) BSS-2 clots. Similar rings of retraction (white arrows) are observed in normal control and BSS blood after 30 min. (C) Flow cytometric analysis of formaldehyde-fixed platelets of PRP from normal (N) blood and PRP from BSS-1 and BSS-2. Surface expression of platelet GPIbα (CD42b) in a (a) normal control. GPIbα is greatly reduced and similar to the isotype control in (b) BSS-1 and (c) BSS-2 platelets. Surface expression of integrin αIIb (CD41) in a (d) normal control, as well as (e) BSS-1 and (f) BSS-2 platelets. Surface expression of integrin β3(CD41) in αIIb/β3 in a (g) normal control, and (h) BSS-1 and (i) BSS-2 platelets. Normal-to-increased expression of integrin αIIb and the αIIb/β3 complex is observed in BSS platelets. (j, k) Isotype control for (j) FITC and (k) PE with normal platelets; BSS-1, BSS-2, GT-1, GT-2, and GT-3 platelets showed very similar controls.

This patient is a 21-year old Caucasian female from a nonconsanguinous parental relationship, diagnosed with BSS at age 7, who has had numerous platelet transfusions throughout her life. At presentation to our facilities, her CBC was normal, except for platelet counts of 28K/µl-50K/µl on three occasions 2–3 months apart (Table 1). Her blood smears displayed large and giant platelets (Figure 1A–a; Table 1), with 72% >4 µm, compared to a normal control (Figure 1A–b; Table 1). Her plasma PT, aPTT, and fibrinogen levels were normal (Table 1), indicating that plasma-based hemostasis was unaffected by her disease.

**Table 1:** CBC and plasma hemostasis parameters.

Human	Fibrinogen (mg/dl)	PT (sec)	aPTT (sec)	Platelets^c^ (×10^3^/µl)	Hemoglobin (g/dl)	WBC (×10^3^/µl)
Controls^a^	272±13	11.5±0.2	32.0±1.1	227±36 24%, 1–2 µm^d^ 76%, 2–4 µm^d^	13.9±0.4	7.1±0.5
BSS-1^b^	264; 261	nd; 11.2	33.5; 29.4	37±7 28%, 1–2 µm^d^ 68%, 2–4 µm^d^ 4%, 4–8 µm^d^	11.5; 11.7	5.8; 6.7
BSS-2^b^	384; 317; 351	10.9; 10.5; 10.4	24.9; 26.3; 26.3	59; 32 0; 14%, 1–2 µm^d^ 17; 5%, 2–4 µm^d^ 66; 38%, 4–8 µm^d^ 17; 43%, 8–12 µm^d^	13.6; 13.6	7.5; 9.3
GT-1^b^	402; 428; 390	9.9; 10.5; 10.2	24.7; 26.6; 25.6	130; 130; 117 3%, 1–2 µm^d^ 53%, 2–4 µm^d^ 43%, 4–6 µm^d^ 2%, 6–8 µm^d^	12.8; 13.5; 13.2	3.4; 3.5; 3.7
GT-2	282	10.8	25.6	202 4%, 1–2 µm^d^ 68%, 2–4 µm^d^ 28%, 4–6 µm^d^	11.2	8.7
GT-3	337	10.2	27.0	224 3%, 1–2 µm^d^ 72%, 2–4 µm^d^ 25%, 4–8 µm^d^	13.7	7.9

a Normal human controls, N = 10.

b Data are from separate blood draws >4 months apart.

c Manual counts with Wright stain.

d Average size distribution of platelets.

#### Patient BSS-2

This patient is a 61-year old Hispanic female diagnosed with BSS at age 2, also frequently administered platelets for clinical procedures, but none within a month of her 3 visits to us that occurred 4 months apart. Her PT, aPTT, and fibrinogen levels were also normal (Table 1). While her WBC count was also normal, her blood sample also showed macrothrombocytopenia (Figure 1A–c; Table 1), with large (4–8 µm) and giant (>8 µm) platelets present, the latter comprising approximately 17% of her total platelets (Table 1).

### Whole Clot Retraction of BSS Blood

Whole blood clot retraction studies, an important property of functional platelets, showed a clear clot retraction ring in control normal blood (Figure 1Bv), as well as for the blood of BSS-1 (Figure 1B–b), reflecting the formation of a thrombus that retracts from the sides of the tube. This property of platelets stimulates healthy wound healing, and suggests normal platelet function in this regard. Whole blood clot retraction for BSS-2 blood was also normal (Figure 1B–c).

### Molecular Genetics of BSS Patients

#### Patient BSS-1

**Figure 2:**
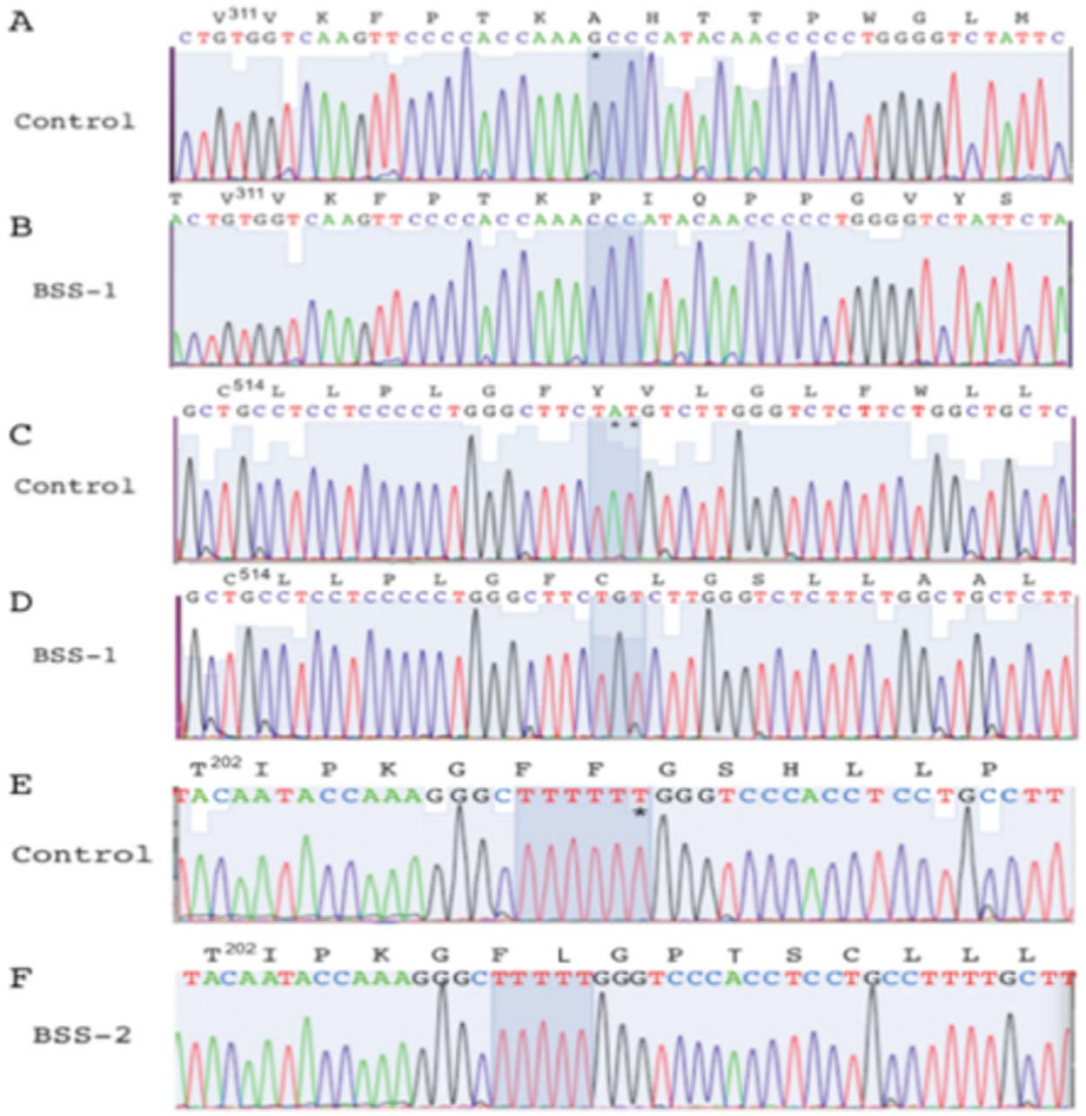
Nucleotide sequence of the mutated regions of *GPIBA* in BSS patients. In each case, the sequence data are displayed for one example PCR clone within the *GPIBA* gene in the genome, along with the readouts of the nucleotides of the translated products. No uncertainties were noted in any clones sequenced in these regions. (A) Normal human *GPIBA* sequence of the region spanning amino acids V^311^–M^326^ of GP1b. (B) The 1^st^ nucleotide (G*) of the codon for A^318^ is deleted in the paternal allele of BSS-1 (A), thus altering the reading frame of the protein. (C) Normal human GP1b sequence of the region spanning amino acids C^514^–L^529^. (D) The 2^nd^ and 3^rd^ nucleotides (A*T*) of the codon for Y^521^ are deleted in the maternal allele of BSS-1 (C), thus altering the reading frame of the protein. (E) Normal human GP1b sequence of the region spanning amino acids T^202^–V^214^. (F) A single T of the group of 6 T residues encoding F^207^–F^208^ of GP1b has been deleted (*) in both alleles of BSS-2 (E), altering the reading frame of the protein. The protein sequences are numbered from M1 of the ORF. The corresponding nucleotide sequences are numbered from the 1^st^ residue of the ATG translation initiation sequence.

Based on our sequence analysis of the *GPIBA* gene of BSS-1, she was determined to be doubly heterozygous for *GPIBA* gene mutations. Of the genomic DNA clones tested, 6 showed deletion of a single nucleotide, G^952^ (numbered from the A^1^TG translation initiation site), altering translation to A^318^P (the Met at the translation initiation site is designated amino acid 1), followed by a frame-shift mutation (Figure 2A, B) and a stop codon beginning at sequence position-334. This *GPIBA* mutation has not been previously identified in BSS. After *in silico* reinsertion of G^952^ and translation, the protein showed two tandem exact 13-amino acid residues repeats, at amino acids 415–440, in the VNTR locus of the protein (allele C of GPIb). Variable numbers (1–4) of these polymorphic tandem exact VTNR repeats in GPIb have been reported [9]. Other polymorphisms have been noted in this protein and have assisted in tracking the genetic pools of the mutations leading to BSS. GPIb, associated with the G^952^ deletion in BSS-1, does not carry the RS polymorphism (T to C) at nucleotide-5 [10], the T^161^M protein polymorphism (Ko) [11], the silent EF polymorphism (C to T) at the 3^rd^ nucleotide of N^258^ [10], or the silent KL polymorphism (A to G) at the 3^rd^ nucleotide of R^358^ [10].

Another group of 9 clones, showed that two nucleotides (A^1562^–T^1563^) of *GPIBA* were deleted (Figure 2C,D), altering translation to Y^521^C in the transmembrane domain of the protein, followed by a frame-shift mutation, resulting in a stop codon at sequence position 602. This latter mutation has been reported in other BSS patients [12], [13]. The translated sequence of the protein, with *in silico* reinsertion of the two deleted nucleotides, showed it also to be allele C in the VNTR locus of GP1b. The gene associated with *GPIBA[ΔA^1562^–T^1563^]* carries the RS and KL polymorphisms, but not the Ko or EF polymorphisms, in common with the previous two patients described with this altered allele [12], [13]. No WT *GPIBA* subclones were found in the DNA of BSS-1.

Blood was obtained from the biological parents of BSS-1 for the study, and both proved to be carriers of one of the two mutations within the *GPIBA* gene of the daughter. Their sequence data were clear in that the paternal *GPIBA* mutant allele possessed *GPIBA[ΔG^952^]* (11/23 subclones), and its translation after reinsertion of G^952^ showed the protein to contain allele C in GPIb, but no other *GPIBA* polymorphism. The father of BSS-1 also carried WT *GPIBA* 12/23 subclones). The maternal *GPIBA* mutant allele possessed *GPIBA[ΔA^1562^–T^1563^]* (3/9 subclones), and, after i*n silico* reinsertion of A^1562^–T^1563^ and translation, the protein also contained allele C of *GPIBA*, and the RS and KL polymorphisms. The maternal genomic DNA also possessed WT *GPIBA* (6/9 subclones). The CBCs of each parent were within normal limits, but the paternal manual platelet count was low at 39K/µl. Blood smears showed large platelets (34% 4–8 µm). The maternal platelet count was 174K/µl and platelet sizes were normal (95% 2–4 µm).

#### Patient BSS-2

Biological relatives of BSS-2 were unavailable. Nonetheless, we suspected the presence of a homozygous double deficiency of *GPIBA*, since her parents were 2^nd^ cousins, as reported by BSS-2. Our sequence analysis of her *GPIBA* gene revealed one population of subclones (20 total), with a deletion of one T in a T-rich segment of the DNA, viz., C^618^TTTTTTG in control *GPIBA* and C^618^TTTTTG in the patient sample (Figure 2E,F), a mutation that has not been previously reported in BSS. This change generated a F^208^L mutation in the patient GPIb, followed by a frame-shift mutation in the gene resulting in a stop codon at sequence position 255. Examination of the remainder of the DNA and translated product, after *in silico* reinsertion of the deleted T, showed it to possess the C allele in the VTNR region of *GPIBA*, but not the RS, Ko, EF, or KL polymorphisms.

We thus conclude that the three allelic mutated *GPIBA* genes that are present in BSS-1 and BSS-2 originated from different founder lines, and BSS-1 represents an extremely rare case of a double heterozygote for two different GPIb inactivating mutations, resulting in BSS. There is no evidence of consanguinity in the family of BSS-1. Thus, this genetic trait resulted from a chance breeding of two different founder lines. To our knowledge, only one other double heterozygote for a GP1b-deficient BSS patient has been fully characterized, and contains separate allelic protein mutations of [C^81^R] (renumbered according to our system for consistency) and [W^527^stop] [14]. These published mutations differ from those described herein. On the other hand, BSS-2 is homogyzous for *GPIBA* inactivating mutations, also resulting in BSS, that originated from a consanguineous breeding of a single founder line, a more common occurrence in BSS.

### FCA of BSS Platelets

PRP isolated from a normal control (N) showed the presence of the platelet surface receptors, GP1bα (Figure 1C-a), integrin αIIb (Figure 1C-d), and integrin β3 in αIIb/β3 (Figure 1C-g). On the other hand, PRP from BSS-1 and BSS-2 demonstrated the absence GP1bα (Figure 1C-b and Figure 1C-c). BSS-1 showed intact-to-increased amounts of integrins αIIb (Figure 1C-e) and β3 in αIIb/β3 (Figure 1C-h), and BSS-2 demonstrated near normal levels (>75%) of αIIb (Figure 1C-f) and β3 in αIIb/β3 (Figure 1C-i). Thus, the interaction of VWF with platelets is adversely affected by the loss of GP1bα, but contains both protein components of the fibrinogen receptor, αIIb/β3.

### GT Patients

Three of these very rare patients were studied.

#### Patient GT-1

This 61-year old male, diagnosed with GT in the mid-1960s, is the offspring of nonconsanguineous parents. Only patient blood was available, since his parents are deceased and there are no siblings. Plasma-based coagulation assays for GT-1 were found to be within the normal range, although his fibrinogen level was at the high end of normal (Table 1). Platelet counts of GT-1 were at the lower limit of normal and, by manual counts, the size distributions of GT-1 platelets had components (45%) that were larger than normal (Table 1). The hemoglobin concentration of GT-1 was normal, while the WBC count was low (Table 1).

#### Patient GT-2

This patient is a female offspring of nonconsanguineous parents who was diagnosed with GT at 4 weeks of age. She was 20 years of age upon arrival to our facilities, accompanied by her biological mother (GT-2M), who did not show outward symptoms of GT, and did not report a history of the disease. GT-2M provided blood for this study. Plasma-based coagulation assays on GT-2 were found to be normal, as were platelet and WBC counts (Table 1). The size distribution of the platelets of GT-2 were similar to GT-1 and had a small population of large platelets (Table 1).

#### Patient GT-3

This 16-year old Caucasian female was diagnosed with GT at age 3 years because of frequent and severe nosebleeds. She also has a history of bruising very easily. This patient has had one platelet transfusion because of nosebleeds (age uncertain). At various times, GT-3 has been administered rFVIIa, amicar, and tranexamic acid for nosebleeds. Apparently, she was provided rFVIIa elsewhere for self-administration when needed. She is taking low dose estrogen and reports recent normal menstrual cycles. Plasma coagulation parameters, as well as CBCs, were normal (Table 1) and her cells showed a small population of slightly larger platelets >4 µm. GT-3 was accompanied to our facilities by her asymptomatic mother (GT-3M), who also volunteered blood for this study.

### Molecular Genetics of GT Patients

#### Patient GT-1

Genetic analyses had not been previously performed on patient GT-1. Thus, nucleotide sequences of all exons of the *ITGA2B* (integrin αIIb) and *ITGB3* (integrin β3) were determined on this patient. In total, the primers designed provided amplicons covering all exons and splice sites in both genes and about 50% of the introns. These sequences were compared to those of *ITGA2B* and *ITGB3* in the GenBank database.

**Figure 3:**
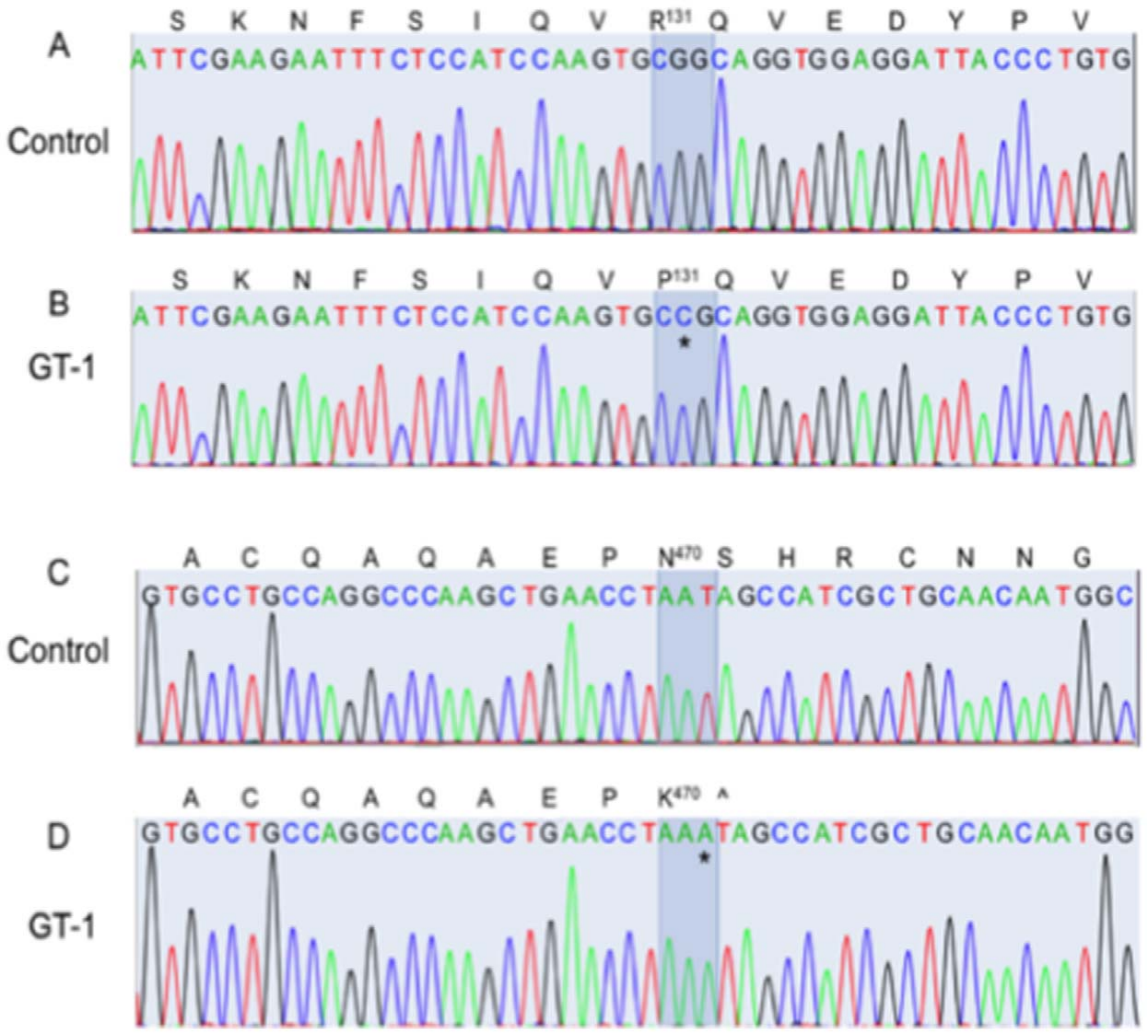
Nucleotide sequence of the mutated regions of the *ITGA2B* and *ITGB3* integrin genes in GT patients. (A–D) GT-1. In each case, the sequence data are displayed for one example PCR clone within the *ITGB3* gene in the genome, along with the readouts of the nucleotides of the translated products. No uncertainties were noted in any clones sequenced in these regions. The *ITGA2B* gene did not show mutations from WT *ITGA2B* in any region of the gene. (A) Normal human *ITGB3* sequence of the region coding for amino acids S^123^–V^138^ of WT αIIb. (B) The 2^nd^ nucleotide (G) of the codon for R^131^ is mutated from G to C(*) in GT-1, thus altering R^131^ to P^131^ in the translated product. (C) Normal human *ITGB3* sequence of the region spanning amino acids A^462^–G^477^. (D) An insertion mutation of an A in the 3^rd^ nucleotide (*) of the codon for N^470^ alters this amino acid to K^470^ and shifts the reading frame such that a stop codon (∧) in the *ITGB3* gene of GT-1 replaces the WT amino acid, S^471^. All protein sequences are numbered from Met^1^ of the ORF of the protein. All corresponding nucleotide sequences are numbered from the 1^st^ residue of the ATG translation initiation sequence.

We did not find mutations in *ITGA2B* exons. As with WT *ITGA2B*, introns 5 and 8 were flanked by the GC-AG dinucleotide donor and acceptor sequences at their 5′ and 3′ termini, respectively. The GC-AG splice site is the major (∼1%) variant of the standard GT-AG (99%) sequences found at intron flanking positions, and is recognized by the spliceosome [15]. Thus, *ITGA2B* appears genetically intact in this patient. The *ITGB3* gene showed two high frequency mutations in its exons in the patient samples. In one, a [G^30,612^C] alteration (Figure 3A, B) occurred (numbered from the A^1^TG translation initiation site) located in exon 4, which is the 2^nd^ nucleotide of the DNA codon (CGG) for R^131^ in WT-β3 integrin (the Met at the translation initiation site is amino acid 1). This mutation yields the DNA codon, CCG, translating to P^131^, thus providing the mutated protein, integrin β3[R^131^P]. This same change occurred in 6/11 subclones sequenced, suggesting that this mutation is present on one allele of the patient. A total of 5/11 subclones showed the WT codon for amino acid R^131^ at this location, thus demonstrating that the β3 is heterozygous for the [R^131^P] mutation, which likely occurred from one parent. All introns were flanked by the standard consensus dinucleotide sequences, GT-AG, at the 5′ and 3′ termini of the introns, respectively, in at least 8/8 subclones sequenced.

A second mutation was found in another location of the *ITGB3* gene of GT-1, specifically insertion of an A^38,427^ (Figure 3C, D), located in exon 10. This mutation altered the WT-AAT codon to AAA, providing the mutation, [N^470^K], and altering the reading frame such that a [S^471^stop] occurred in the β3 protein. A total of 5/11 subclones contained this mutation, while the others displayed the WT amino acid, S^471^, at this position. This mutation most likely occurred in the other parental allele. No other alterations occurred in the exons or intron splice junctions in *ITGB3* in this patient.

Thus, the complete genetic data show that GT-1 is doubly heterozygous in the *ITGB3* alleles, with one parent providing the [R^131^P] mutation, and the other transmitting the [N^470^S^471^]/[K^471^stop] mutations. Since GT is an autosomal recessive disorder, it would appear that either mutation, in itself, would inactivate the αIIb/β3 complex, thus severely attenuating fibrin(ogen)-dependent platelet aggregation and platelet contributions to stable clot formation, as is seen experimentally. We conclude that patient GT-1 is a double heterozygote of two β3 integrin inactivating mutations.

#### Patient GT-2

The genetics of GT-2 have been previously studied and a 6 residue deletion (G^1392^TAGAC; numbered from A^1^TG) was found in exon 13 of the *ITG2AB* gene [16], corresponding to a 2 amino acid (V^453^D^454^; numbered from M^1^) deletion in integrin αIIb protein. This deletion eliminated two highly conserved amino acids from the fourth Ca^2+^ binding domain of αIIb, resulting in diminished surface expression of αIIb and a loss of the native conformation of the heterodimeric αIIb/β3.

#### Patient GT-3

Molecular genetic testing was not performed on GT-3 or GT-3M.

### Deposition of Nucleotide Sequences

Nucleotide sequences of the abnormal maternal (BSS-1M) and paternal (BSS-1F) *GP1BA* alleles, both of which are inherited by BSS-1, the homozygous abnormal *GP1BA* alleles of BSS-2, the *ITGB3* abnormal alleles in patient GT-1, and one abnormal *ITG2AB* allele of GT-2 have been deposited in the GenBank database. The accession numbers for the 5 sequences are KC120774-KC120778.

### Clot Retraction Studies of GT Patients

**Figure 4:**
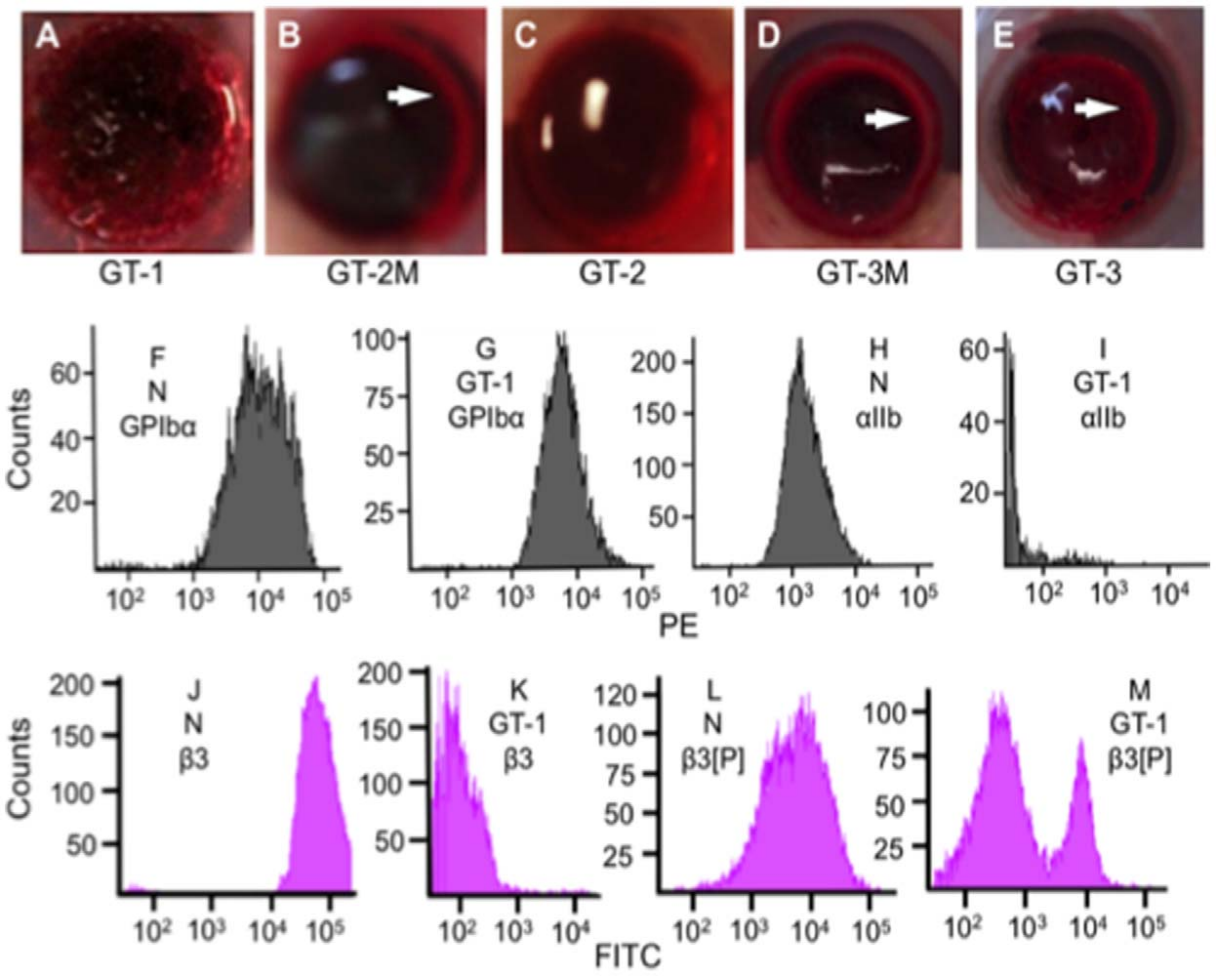
Platelets of GT patients. Whole blood clot retraction of (A) GT-1, (B) GT-2M, and (C) GT-2, (D) GT-3M, and (E) GT-3 clots. A ring of retraction (white arrows) is observed in GT-2M and GT-3M clots, whereas the GT-1, GT-2, and GT-3 specimens displayed much weaker retraction at 30 min. (F-M) Flow cytometric analysis of normal and GT-1 PRP. (F, G) Surface expression of GP1bα (CD42b) in (F) normal and (G) GT-1 platelets show similar expression levels of GP1bα. (H, I) Surface expression of integrin αIIb (CD41) in (H) normal and (I) GT-1 platelets show reduced expression of CD41 in GT-1 platelets, likely owing to the reduced levels of β3 and thereby reduced amount of surface αIIb/β3 complexes. (J) Surface expression of integrin β3 (CD61) in normal platelets from PRP shows one peak demonstrating the presence of integrin β3 in the αIIb/β3 complex. (K) Surface expression of platelet β3 of αIIb/β3 in GT-1 PRP shows a lack of reactivity of platelets with this antibody. (L) Surface expression of integrin β3[740–769] (β3[P]) in normal PRP. (M). Surface expression of integrin β3[740–769] (β3[P]) in GT-1 PRP. The downfield peak in (M) is similar to isotype controls and likely corresponds to the protein expressed by the truncated allele of integrin β3 in αIIb/β3 in this patient. The smaller peak in (M) to the right shows a reactivity with the full-length allele of integrin β3 in this complex, which harbors the [R131P] mutation but expresses the [740–769] peptide region of the protein.

Clot retraction performed on the GT specimens were variable. GT-1 (Figure 4A), showed no clot retraction after 30 min and instead revealed a homogeneous image without any distinctive contractile movement along the transverse plane, demonstrating impaired platelet function. Clot retraction of GT-2M (Figure 4B) whole blood was normal, while GT-2 (Figure 4C) showed weaker retraction tendencies (∼2% of WT) than WT blood. The blood of GT-3M (Figure 4D) was normal in this regard, and that of GT-3 (Figure 4E) showed ∼50% of GT-3M retraction after 30 min. The weaker retraction properties of GT blood are due to the genetic functional attenuation of receptor αIIb/β3, which mediates platelet contraction and aggregation [16]. These three patients appear to have variable disease severity, as measured by clot retraction properties, with GT-1 the most profound example of GT, and GT-3 the least. This diversity in properties is likely due to the wide array of possible mutations present in αIIb and β3.

### FCA of the GT Platelets

#### Patient GT-1

PRP specimens from GT-1 were analyzed by FCA. A comparison of the FCA graphical representations of normal (N) PRP (Figure 4F) and GT-1 PRP (Figure 4G) shows a normal level of GP1bα. However, when compared to a normal control (Figure 4H), GT-1 displayed a near complete loss of reactivity of the antibody to integrin αIIb (Figure 4I), and was comparable in strength to the isotype IgG_1_ control. Upon examination of integrin β3 with a conformational antibody to β3 in the αIIb/β3 complex, less than 5% of normal β3 (Figure 4J) was evidenced in the platelets of GT-1 (Figure 4K). Another CD61 antibody, that recognizes the peptide region encompassing amino acids [740–769], reacts with control WT platelets (Figure 4L), but shows mixed reactivity in GT patients (Figure 4M). Since β3 in GT-2 platelets has a stop codon at amino acid 471, the β3 translated by this allelic DNA would not be expected to react with this antibody. This is borne out, as seen by one population in Figure 4M that is similar to the isotype control. However, the other allele of GT-1 platelets would translate a full-length mutated β3 protein, which, if stably produced, would react with this antibody, as seen in the second upfield population of Figure 4M. The results with these two antibodies are in agreement with the DNA sequencing that showed that this patient generates a mutated full-length β3 on one allele and a truncated allele on the other, but the conformation of this protein is altered from the WT protein.

#### Patient GT-2

**Figure 5:**
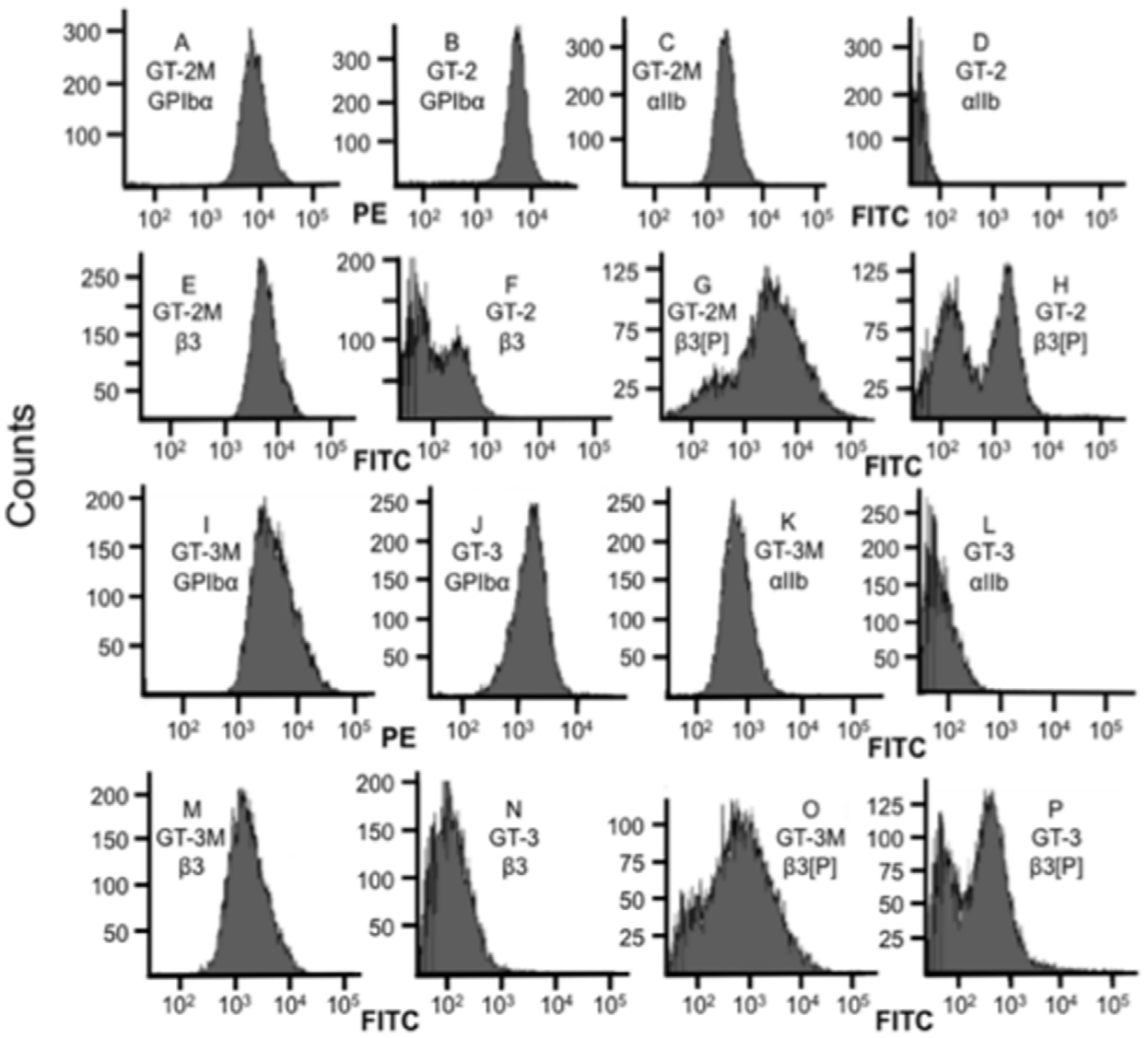
FCA of platelets from PRP of symptomatic GT-2 and GT-3 patient blood, and their heterozygous mothers (GT-2M and GT-3M, respectively). (A–H) Analysis on the PRP of GT-2 and her heterogyzous mother (GT-2M). Normal expression of GPIbα is seen in (A) GT-2M and (B) GT-2. Integrin βIIb is expressed on platelets of (C) GT-2M, but not (<5%) expressed on the platelets of (D) GT-2. Integrin β3 in the αIIb/β3 complex is expressed on platelets of (E) GT-2M, but not (<5%) expressed on the platelets of (F) GT-2. The amino-terminal peptide [residues 740–769] of β3 (β3[P]) is expressed on the platelets of (G) GT-2M and partly expressed (<10%) on platelets of (H) GT-2 (upfield peak), with another peak (downfield) of weak expression on GT-2. (I, J) Expression of GPIbα is similar in GT-3M and GT-3, whereas (K, L) expression of αIIb is greatly attenuated in GT-3, compared to GT-3M. (M, N) Similarly, integrin β3 in αIIb/β3 displays greatly reduced expression in GT-3 compared to GT-3M. (O, P) The C-terminal peptide region of β3 is recognized in GT-3M, and only partly recognized GT-3 (likely maternal allele), suggesting that a truncation of β3 is present in one allele (likely paternal) in the patient.

The platelets in the PRP of patient GT-2 also displayed normal levels of GP1b, when compared to samples taken from GT-2M, her biological mother (Figure 5A, B). However, reactivity to anti-integrin αIIb was very weak compared to her mother (Figure 5C, D). This suggests a significant loss of the surface native αIIb epitope in GT-2 platelets. Similarly, a loss in reactivity of the conformational epitope to anti-CD61 (β3) is noted in the αIIb/β3 complex on the platelet surface of GT-2, as compared to the platelets of GT-2M (Figure 5E, F). With this antibody, one population displaying no antibody binding was observed, and a second upfield population displaying very weak antibody binding was also seen (Figure 5E, F). Similarly, approximately 50% loss in reactivity to the linear C-terminal reactive antibody of β3 (recognizing 740–769 amino acids of β3) was observed in GT-2 (Figure 5H), when compared to reactivity of the GT-2M platelets (Figure 5G). However, the one subset of platelets (50%) in GT-2 remains, with a native reactivity to the linear C-terminus of β3, as observed by the upfield peak in Figure 5H. These data demonstrate that full-length β3 is present in the platelets of patient GT-2.

#### Patient GT-3

The platelets of GT-3M and GT-3 essentially showed the same FCA properties (Figure 5I-P) with these antibodies when compared to GT-2M and GT-2, with only qualitative differences between the paired samples.

### TEG Analysis of Normal Human Whole Blood Clotting

**Figure 6:**
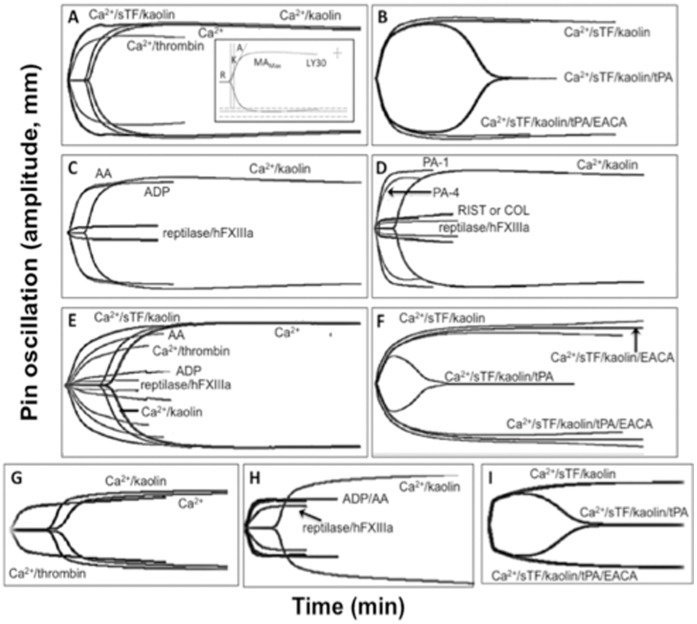
Representative TEG traces of human whole blood. (A–D) Representative normal control. Samples were collected in citrate-containing plastic tubes for additions of (A) Ca^2+^, Ca^2+^/kaolin, Ca^2+^/sTF/kaolin, or Ca^2+^/thrombin; (B) Ca^2+^/sTF/kaolin/tPA or Ca^2+^/sTF/kaolin/tPA/EACA, compared to the trace with Ca^2+^/sTF/kaolin/tPA. Samples were collected in heparin-containing plastic tubes and used for platelet stimulation of the MA_F_ generated by (C) reptilase/hFXIIIa with AA or ADP; or (D) COL, RIST, PA-1, or PA-4. The MA_max_ obtained (from A) with Ca^2+^/kaolin was used as the 100% reference to compare all agonists for calculation of the degree of agonist stimulation. The inset in Figure 6A displays the parameters that were calculated by the software. (E-I) Similar measurements for (E, F) BSS-2 and (G-I) GT-1 whole blood, collected under the same conditions as normal control blood.

We considered the values of R, K_20_, A, MA_max_, LY_30_, and LY_60_ (Figure 6A, inset) in recalcified plasma of control samples (Figure 6A; Table 2), as basal measures of overall normal hemostasis balance in whole blood. It is seen that the fibrin formed in heparin/reptilase/hFXIIIa (Figure 6C), absent thrombin-induced platelet involvement, has very low viscoelastic strength, as reflected by the MA_max_ value (Figure 6C; Table 2). Thus, MA_F_ is minimal (Figure 6C; Table 2). Additionally, in the case of a fibrinogen deficiency, activated platelets, alone, do not provide a thrombus that is of measurable viscoelastic strength, with MA values being essentially zero [17]. These data suggest that both functional platelets and fibrinogen are critical to development of a stable viscoelastic thrombus. Regarding fibrinolysis, the activation state (-tPA) or potential activation state (+tPA ± EACA) of the fibrinolytic system, as reflected by LY_30_ and/or LY_60_, are employed as diagnostic measures of pathological fibrinolysis in individuals, while also reporting the overall competency of the fibrinolytic system. The values for normal blood are provided in Table 2, as obtained from traces, such as in Figure 6B.

**Table 2:** Whole blood TEG parameters.

Human	R (min)	K_20_ (min)	A (degrees)	MA_max_ (mm)	LY_30_ (%)	LY_60_ (%)
**Controls** a Ca^2+^ Ca^2+^/kaolin Ca^2+^/sTF/kaolin Ca^2+^/sTF/kaolin/htPA Ca^2+^/thrombin Reptilase/hFXIIIa	9.6±0.6 7.3±0.4 0.8±0.1 0.6±0.1 0.8±0.1 1.2±3	2.6±0.2 2.1±0.2 1.2±0.2 1.5±0.2 2.8±0.3 nd	56±3 59±3 75±2 72±2 60±4 nd	62±2 63±2 64±2 62±3 45±4 5.4±0.8	0 0 2.5±1.1 48±7 0 nd	<2<2 5.0±0.7 75±3<2 nd
**BSS-1** c Ca^2+^ Ca^2+^/kaolin Ca^2+^/sTF/kaolin Ca^2+^/sTF/kaolin/htPA Ca^2+^/thrombin Reptilase/hFXIIIa	10.6; 18 7.7; 14.9 0.8; 1.1 0.3 0.8; 0.8 0.8; 2.2	5.8; 4.4 2.3; 3.4 2.5; 2.7 4.8 6.2; 5.4 nd	34; 41 57; 47 71; 63 71 37; 40 16; 17	54; 59 63; 60 62; 57 26 46; 49 8; 4	0 0 0 49 0 0	<2<2<2 74<2 0
**BSS-2** c Ca^2+^ Ca^2+^/kaolin Ca^2+^/sTF/kaolin Ca^2+^/sTF/kaolin/htPACa^2+^/thrombin Reptilase/hFXIIIa	11; 5.7 8.6; 5.4 0.7; 0.8 0.2; 0.7 0.8; 0.6 0.9; 0.5	2.7; 1.7 2.5; 1.7 1.1; 1.6 1.7; 1.6 3.5; 2.2 nd; nd	56; 68 58; 54 78; 73 69; 72 53; 65 27; 55	71; 76 75; 77 74; 69 37; 37 63; 74 7; 10	0; 0 0; 0 0; 0 77; 87 0; 0 nd; nd	<2; <2<2; <2<2; <2 74; 100<2; <2 nd; nd
**GT-1** c Ca^2+^ Ca^2+^/kaolin Ca^2+^/sTF/kaolin Ca^2+^/sTF/kaolin/htPACa^2+^/thrombin Reptilase/hFXIIIa	9.0; 12 6.9; 8.6 0.8; 1.0 0.3; 0.6 0.5; 1.2 0.6; 0.5	3.4; 9.8 2.5; 3.5 1.8; 4.9 2.1 nd; nd nd; nd	52; 35 39; 23 77; 62 75; 72 55; 35 51; 67	25; 21 32; 28 30; 27 23; 15 18; 17 15; 15	0; 0 0; 0 0; 0 72; 88 0; 0 nd; nd	0; 0 0; 0 0; 0 86; 93 0; 0 nd; nd
**GT-2** Ca^2+^ Ca^2+^/kaolin Ca^2+^/sTF/kaolin Ca^2+^/sTF/kaolin/htPACa^2+^/thrombin Reptilase/hFXIIIa	10 5.1 0.9 0.9 1.1 0.7	6 4.6 5.4 5.7 nd nd	36 51 64 65 32 57	30 34 36 25 13 10	0 0 0 81 0 nd	0 0 0 91 0 nd
**GT-3** Ca^2+^ Ca^2+^/kaolin Ca^2+^/sTF/kaolin Ca^2+^/sTF/kaolin/htPACa^2+^/thrombin Reptilase/hFXIIIa	4.8 3.8 0.9 0.6 0.8 0.3	1.8 1.6 1.8 1.6 4.5 nd	66 67 69 75 46 53	67 66 64 27 47 21	0.2 0.5 1.1 95 0 nd	nd nd nd nd nd nd

a Normal human controls, N = 10.

b [htPA] = 200 ng/ml.

c Data are from separate blood draws >4 months apart.

nd, not determined.

These concepts were expanded by analyses of rates, levels, and strength of thrombus formation after additions of other components of the clotting cascade to blood samples. R-values were greatly decreased upon addition of Ca^2+^/thrombin, and thrombi of high viscoelastic strength were formed (Figure 6A; Table 2). In this case, the MA_max_ achieved was not optimal since the high thrombin level initially present likely affected the strength of the thrombus, possibly resulting from the alteration of strand assembly dynamics of the fibrin via the more rapid formation of fibrin by exogenous thrombin [18], [19]. Thrombi formed with Ca^2+^/kaolin provided thromboelastogram-derived values that were only slightly altered from those of Ca^2+^, alone (Figure 6A; Table 2). This shows that the intrinsic pathway is operating to near maximal capacity in maintenance of basal hemostasis and does not accelerate or stabilize thrombi upon further addition of kaolin. However, addition of Ca^2+^/sTF/kaolin to blood resulted in much lowered R and K_20_ values and provided a high MA_max_ (Figure 6A, Table 2), demonstrating that the TF pathway is likely not highly functional in basal hemostasis and likely provides a rapid response mechanism to injury. Lastly, unstimulated LY_30_ and LY_60_ values were very low in control blood (Figure 6B, Table 2), suggesting a minor degree of basal fibrinolysis in healthy individuals. With thrombi rapidly formed by Ca^2+^/sTF/kaolin, inclusion of htPA (200 ng/ml) to human blood resulted in complete lysis (Figure 6B). Further, addition of EACA to Ca^2+^/sTF/kaolin/htPA completely inhibited the lysis of whole blood thrombi (Figure 6B).

### Platelets Provide Viscoelastic Strength to the Human Thrombus in Normal Plasma

**Figure 7:**
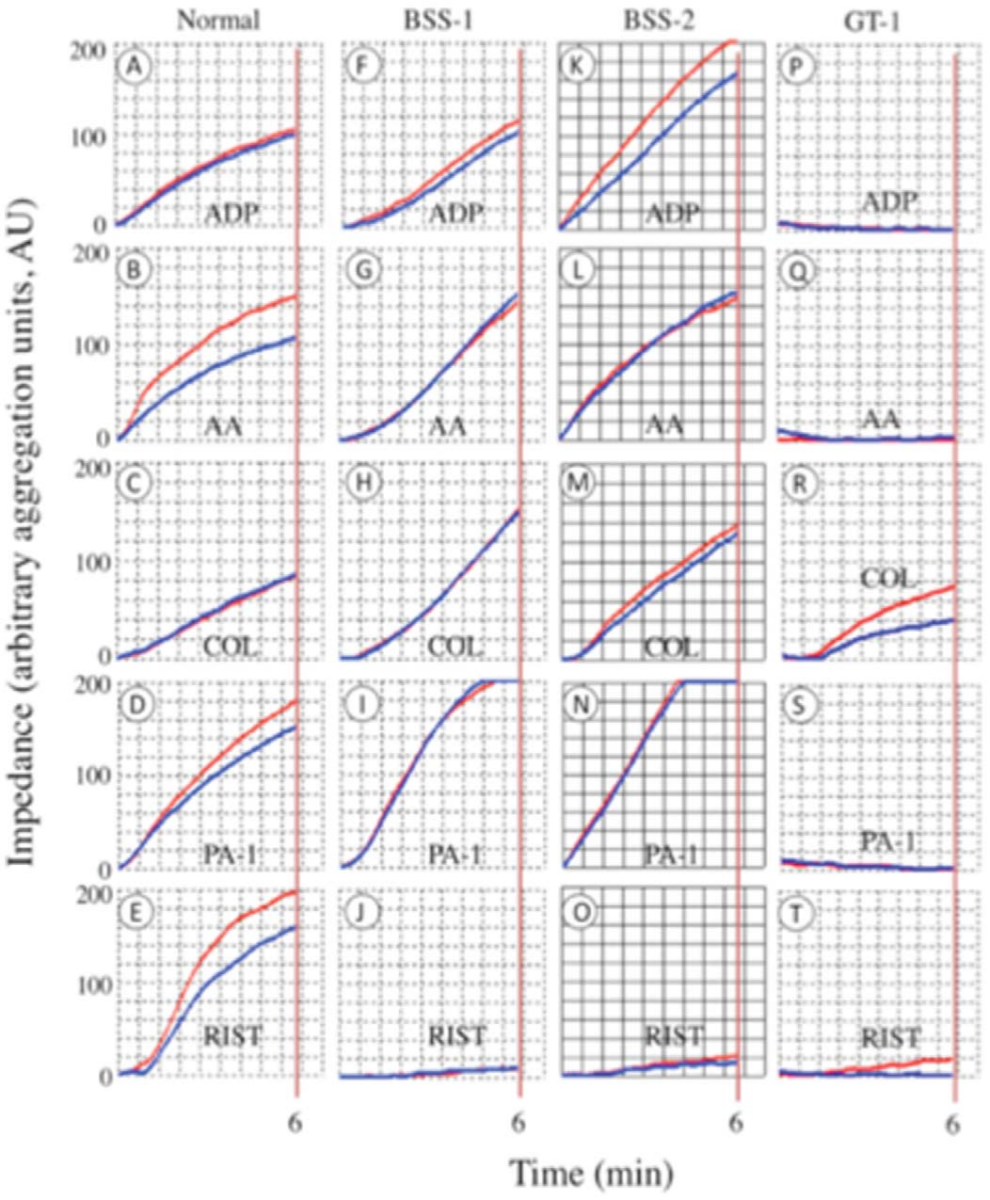
Human whole blood platelet aggregation measurements. The increase in impedance of platelets aggregated on multiplate sensors and transformed by software into arbitrary aggregation units is plotted against time. Curves are generated for 6 min and the areas under the curve, from 0–6 min, are calculated by the software as a measure of platelet aggregation. Two independent runs were generated for each agonist (blue and red curves in each panel) and the mean of the areas was employed. The agonists are listed on each panel. Data were collected on normal human whole blood, and on BSS-1, BSS-2, and GT-1 blood.

In the case of TEG-based platelet functional analysis of normal human whole blood, the MA_A_ is measured in heparin/reptilase/hFXIIIa blood after addition of a platelet activator (e.g., ADP, AA, RIST, COL, PA-1, PA-4). In these experiments, fibrin is formed by reptilase, which is very similar to that formed by thrombin [20], and crosslinked by exogenous FXIIIa, and any thrombin generated is inhibited by heparin/antithrombin III. Different platelet activators are then added, and the MA_max_ determined. The MA ratios (with the MA_F_ subtracted), with and without platelet activators, allowed assessment of the effectiveness of the individual platelet agonists to stimulate the assembly of strong whole blood thrombi. The data obtained for the human controls (Figure 6C, D; Table 3) show that the agonists, ADP and AA (Figure 6C), as well as the PAR-1 and PAR-4 agonist peptides, PA-1, and PA-4, respectively (Figure 6D), result in thrombi of strong viscoelasticity. However, RIST and COL do not allow thrombi of high strength to be formed (Figure 6D) at concentrations of each that effectively aggregate platelets in whole blood (Figure 7C,E, Table 4), when aggregation is measured independently of thrombus formation.

**Table 3:** Platelet mapping by TEG.

Human	ADP^c^ (%) 2 µM^d^	AA^c^ (%) 1 mM^d^	RIST^c^ (%) 0.3 mg/ml^d^	COL^c^ (%) 3.2 µg/ml^d^	PA-1^c^ (%) 31 µM^d^	PA-4^c^ (%) 663 µM^d^
Controls^a^	78±4	99±2	4±2	5±2	84±6	73±6
BSS-1^b^	22; 36	62; 66	−6.4; 0	−4.4; 1	48	32
BSS-2^b^	20; 5	92; 90	−1.3; 0	0.5; 0	84; 60	64; 71
GT-1^b^	22; 0	26; 0	7; 0	0; 3	14; 0	0; 0
GT-2	0	37	0	0	20	0
GT-3	68	0	0	21	69	76

a Normal human controls, N = 10.

b Data are from separate blood draws >3 months apart.

c The stimulation by each of these agents with respect to the MA of Ca^2+^/kaolin ( = 100%).

d Final concentration in the reaction vessel.

**Table 4:** Impedance-based platelet aggregometry (AU) of the study groups^a^.

Human^b^	ADP 6.5 µM^d^	AA 0.5 mM^d^	RIST 0.88 mg/ml^d^	COL 3.2 µg/ml^d^	PAR-1 32 µM^d^	PAR-4 663 µM^d^
Controls^b^	125±10	143±10	146±8	136±10	195±47	213±8
BSS-1^c^	109; 93; 92	147; 148; 121	10; 0; 8	150; 149; 84	219; 182; 165	161; 153
BSS-2^c^	187; 146; 168	150; 137; 150	18; 37; 37	134; 60; 124	272; 199	162; 171
GT-1^c^	0; 0	0; 0	10; 0	57; 85	0; 5	0
GT-2	0	105	74	163	5	5
GT-3	29	43	95	69	42	39

a Two analyses were performed for each agonist in all cases. Data are reported as the mean value of the areas under the respective curves.

b Normal human controls, N = 10.

c Data are from separate blood >4 months apart.

d Final concentration in the reaction vessel.

### TEG and Platelet Aggregometry Analyses of BSS and GT Whole Blood Clotting

There are differences between whole blood thrombus stability in normal and BSS-derived whole blood, but some differences between the two BSS patients are also seen (Table 2), probably due to the disparate nature of the deficiencies in each case. Upon recalcification of citrated blood, the R and K_20_ are slightly prolonged in BSS-1 samples, and less so in BSS-2 blood (Figure 6E; Table 2). This indicates a defective level of functional thrombin in these patients, undoubtedly due to the thrombocytopenia and the large platelet surface areas. Both of these factors would attenuate the levels and availability of the prothrombinase complex on platelets. This hypothesis is fortified by the large decrease in R after addition of Ca^2+^/thrombin (Figure 6E, Table 2). K_20_ is not affected in this situation because new thrombin is not formed at a more rapid rate when Ca^2+^/thrombin is added. However, the R and K_20_ of BSS whole blood (BSS-2 is shown as the example) are greatly decreased after addition of Ca^2+^/sTF/kaolin (Figure 6E, Table 2). As with normal whole blood, addition of Ca^2+^/kaolin did not remarkably alter these TEG parameters, again showing that the intrinsic system functions to maintain basal hemostasis in the individual. Thus, despite low platelet counts and large platelets, strong viscoelastic thrombi are formed with BSS platelets in whole blood. As with normal controls, no unstimulated fibrinolysis occurred in BSS blood (Figure 6F, Table 2), but addition of htPA (200 ng/ml) to BSS blood resulted in complete lysis, a process also nearly completely inhibited by EACA (Figure 6F).

Platelet functionality in whole blood thrombus formation, as analyzed by TEG, is remarkable in that ADP stimulation of BSS platelets is defective with regard to generating strong viscoelastic thrombi with fibrin (Figure 6E, Table 3), compared to control blood, despite the finding that ADP leads to near normal impedance-based aggregation of BSS platelets (Figure 7A, F, K; Table 4). On the other hand, AA stimulation of the MA_F_ is similar to that of normal blood (Figure 6E; Table 3), as is the impedance-based aggregation of these platelets (Figure 7B, G, L; Table 4). As expected, there was severe attenuation of platelet aggregation with RIST in the BSS patients compared to normal blood (Figure 7E,J,O; Table 4), but other platelet activators tested (AA, COL, PA-1, and PA-4; example provided for PA-1 in Figure D, I, N) appeared quantitatively similar to the blood of normal controls (Table 4).

Regarding GT whole blood clotting, values of R, K_20_, and A in recalcified blood were similar to WT control blood (Figure 6G, H; Table 2). The response of these values to thrombus formation with Ca^2+^/kaolin, Ca^2+^/thrombin, and Ca^2+^/sTF/kaolin were also similar to WT controls. This indicates that platelets were activated by thrombin in GT blood and could assemble into a thrombus. However, as reflected by the substantially lower MA_max_ value (Figure 6G, Table 2), platelet/fibrin interactions were affected and assemble into a substantially weaker thrombus, whether the platelets were activated by thrombin (Figure 6G, Table 2); ADP, AA (Figure 6H); or ristocetin, collagen, PA-1, or PA-4 (not shown). In separate aggregation studies, of all the platelet activators examined, only collagen weakly stimulated aggregation, likely via binding to a separate receptor on platelets. Ristocetin did not function in this manner in aggregation assays, despite the functional presence of GP1b (Figure 7, Table 4).

The thrombi formed in the GT whole blood did not show spontaneous clot lysis, but did display very strong lysis with htPA (Figure 6I, Table 2), in a process that was effectively inhibited by EACA. Perhaps the more rapid lysis of the clots in GT blood displayed at 30 min (Table 2) is due to the weakened clots formed in this system.

### Discussion and Conclusions

While it is difficult to recapitulate in vivo hemostasis in ex vivo models, nonetheless much has been learned that is clinically applicable over decades of efforts in this regard. Studies of hemostasis mechanisms in plasma have focused on the role of soluble factors in fibrin formation. However, it is relevant to expand hemostasis mechanistic studies to whole blood to include the role of platelets in clinical consideration of the hemostasis status of patients. A manner of understanding the features of platelets needed to form stable thrombi is to study whole blood that contains platelets with known deficiencies. BSS patients afford one such opportunity. In this case, the contributions of GP1b in whole blood thrombus formation can be studied in the absence of high shear. However, the numbers of these patients are very limited (1∶1,000,000), and only ∼100 cases have been confirmed in the literature. Fortunately, we were able to access two BSS patients for this work. Deficiencies in human GP1b, both genetic and acquired, are found in BSS [21], cardiopulmonary bypass (CPB) [22] surgery, and surgical trauma [23], and in targeted transgenic mice [24], and lead to bleeding diatheses, presumably due to defects in the binding of platelets to the subendothelium. Whether such a deficiency would alter rates of thrombus formation and ultimate strength of the thrombus in whole blood with exogenous soluble subendothelium components, was not known, and is one contribution from this work.

Severe attenuation, of human GP1b expression, as in the case of BSS, leads to macrothrombocytopenia, but this has minimal effects on overall plasma-based coagulation markers. In humans, TEG-based whole blood coagulation parameters are also not substantially affected whether thrombin or a direct pharmacological agonist of downstream PAR-1 activation is employed as the overall activator of platelets. However, when thrombin is blocked and other platelet activators are used to induce thrombus assembly in the presence of fibrin, the results are somewhat surprising. In normal human plasma, RIST and COL aggregate and probably activate platelets, but stable viscoelastic thrombi are minimally formed. On the other hand, ADP and AA both aggregate control platelets and allow normal thrombus assembly. In human BSS blood, AA functions normally, but the effects of ADP on thrombus assembly and/or stability are greatly attenuated compared to normal controls. The fact that direct activation of PAR-1 by PA-1 reverses the weakened stimulation by ADP of the MA_F_ in BSS whole blood links ADP stimulation of thrombi of high viscoelastic strength to PAR-1 activation.

Confirmation of the importance of the fibrin(ogen)-binding integrin, αIIb/β3, in assembling a thrombus has been obtained by examination of the blood of human GT patients. GT-1 is shown as the example, an individual who possessed a rare double heterozygous GP1b mutation that inactivated this receptor. This GT blood did not show effective platelet aggregation with a series of platelet activators, except weakly in the case of collagen, and did not assemble with fibrin into a strong viscoelastic clot. Thus, thromboelastograms obtained on patients with deficiencies or blocks of αIIb/β3, would manifest in very low MA_max_ values. Qualitatively similar results are seen with 2 other GT patients with different mutations. Thus, while general principles can be established with an array of BSS and GT patients, given the array of possible mutations that lead to BSS and GT, it is suggested that each patient is examined individually.

Overall, the results demonstrate that human platelet activation, as measured by the ability of platelets to aggregate, does not necessarily translate to the assembly of a thrombus of high viscoelastic strength, and further suggests that the platelet receptors engaged for activation are mechanistically related to the nature of the platelet-fibrin assembly that is measured by clot strength. The contribution of platelet defects, and specifically that of attenuation of GP1b expression [25], to whole blood hemostasis has been a long-standing consideration in on-pump CPB surgery, where TEG analysis on whole blood is employed to predict post-surgical bleeding [26], [27], and off-pump coronary artery bypass surgery led to lower platelet activation defects than on-pump surgery [28].

These factors may also be applicable to the coagulopathy of traumatic brain injury [29]–[34], wherein ADP stimulation of platelets to assemble into a thrombus is diminished, as is expression of platelet surface GP1b [23], but AA stimulation of thrombus formation is affected to a lesser degree [35]. As with the high mechanical shear forces to which platelets are exposed in CPB, high tissue shear forces are also present in certain types of severe TBI, e.g., inertial injury, and can result in excess vWF-dependent activation and possible shedding of the ectodomain of GP1b [36], [37]. Thus, platelets might be generated in severe TBI that are GP1bα deficient, as in BSS. Therefore, BSS platelets are not only critical to understand the mechanistic role of GP1bα in platelet aggregation and thrombus strength, but may also model some of the platelet dysfunctions in the hypocoagulopathy of TBI and CPB, and other maladies.

The results of this study segregate platelet activation, reflected by platelet aggregation, from that represented by assembly into thrombi of high viscoelastic strength, and suggest that the nature of the thrombus formed depends on the receptors and signaling mechanisms that are engaged. ADP stimulation of thrombus formation is a particularly sensitive indicator of the nonsingularity of platelet activation. These findings have implications in treatment of platelet-based coagulopathies, e.g., CPB and TBI, and suggest that platelet counts and platelet aggregation tests, alone, may not sufficiently correlate with effective thrombus formation, and patients, who appear normal in these regards, may still benefit from platelet replacement therapy. More specifically, in the case of coagulopathic patients, thromboelastographic analysis of whole blood thrombus formation and stability is an effective guide for goal-directed administration of blood products [35].
